# *Himar1* Transposon for Efficient Random Mutagenesis in *Aggregatibacter actinomycetemcomitans*

**DOI:** 10.3389/fmicb.2017.01842

**Published:** 2017-09-26

**Authors:** Qinfeng Ding, Kai Soo Tan

**Affiliations:** Faculty of Dentistry, National University of Singapore, Singapore, Singapore

**Keywords:** mutagenesis, conjugation, biofilm, adherence, DNA transposable elements, periodontal disease

## Abstract

*Aggregatibacter actinomycetemcomitans* is the primary etiological agent of aggressive periodontal disease. Identification of novel virulence factors at the genome-wide level is hindered by lack of efficient genetic tools to perform mutagenesis in this organism. The *Himar1* mariner transposon is known to yield a random distribution of insertions in an organism’s genome with requirement for only a TA dinucleotide target and is independent of host-specific factors. However, the utility of this system in *A. actinomycetemcomitans* is unknown. In this study, we found that *Himar1* transposon mutagenesis occurs at a high frequency (×10^-4^), and can be universally applied to wild-type *A. actinomycetemcomitans* strains of serotypes a, b, and c. The *Himar1* transposon inserts were stably inherited in *A. actinomycetemcomitans* transconjugants in the absence of antibiotics. A library of 16,000 mutant colonies of *A. actinomycetemcomitans* was screened for reduced biofilm formation. Mutants with transposon inserts in genes encoding pilus, putative ion transporters, multidrug resistant proteins, transcription regulators and enzymes involved in the synthesis of extracellular polymeric substance, bacterial metabolism and stress response were discovered in this screen. Our results demonstrated the utility of the *Himar1* mutagenesis system as a novel genetic tool for functional genomic analysis in *A. actinomycetemcomitans*.

## Introduction

*Aggregatibacter actinomycetemcomitans*, a gram-negative coccobacillus is strongly associated with localized aggressive periodontal disease which involves rapid loss of alveolar bone in adolescents (Slots et al., 1980; Zambon, 1985; Haubek et al., 2008). This organism is also implicated in non-oral infections such as endocarditis, septicemia, and osteomyelitis (Martin et al., 1967; Meyer and Fives-Taylor, 1998; van Winkelhoff and Slots, 1999). *A. actinomycetemcomitans* expresses numerous virulence factors including leukotoxin, collagenase, and cytolethal distending toxin which contribute to the destruction of periodontium (Shenker et al., 2004; Kachlany, 2010). Even though substantial progress has been made in understanding the virulence of *A. actinomycetemcomitans*, it remains a challenge to study the genetics of the organism’s colonization and persistence in the oral cavity, and identify novel virulence factors due to limited genetic tools available to manipulate *A. actinomycetemcomitans*.

Forward genetics is a powerful tool to identify novel genes critical for virulence. Tn*916*, Tn*5*, Tn*10*, and IS*903* based gene delivery systems have been used to generate mutant libraries in *A. actinomycetemcomitans* which led to discovery of genes essential in virulence and survival of *A. actinomycetemcomitans* (Kolodrubetz and Kraig, 1994; Thomson et al., 1999; Kachlany et al., 2000a; Isaza et al., 2008; Nunes et al., 2016). However, these systems suffer from several drawbacks. The Tn*916* and Tn*5* systems have low efficiency of DNA transposition (≤10^-7^). Although this can be overcome by the use of Tn*10* and IS*903*ϕ*kan* systems which yield higher transposition efficiency, Tn*10* system has preference for hotspots (Halling and Kleckner, 1982). The cryptic *kan* gene of the IS*903*ϕ*kan* system is expressed only when inserted into an expressed gene in the appropriate reading frame to generate a gene fusion with *kan* gene (Thomson et al., 1999). Therefore, it remains challenging to obtain genome wide saturated mutagenesis of *A. actinomycetemcomitans* with the currently available mutagenesis systems. Furthermore, efficiency of these genetic manipulation systems varies considerably among serotypes of *A. actinomycetemcomitans* (Clewell and Gawron-Burke, 1986; Kolodrubetz and Kraig, 1994).

The *mariner* family of transposons, first identified in horn fly, is known to be widespread in nature. The *Himar1 mariner* transposon is one of the two known active *mariner* elements that have been successfully utilized for a number of oral and non-oral bacteria such as *Pseudomonas aeruginosa* (Withers et al., 2014), *Porphyromonas gingivalis* (Klein et al., 2012), and oral streptococci (Nilsson et al., 2014). A key feature that separates these elements from many other transposons is their independence from host-specific factors other than the presence of a TA dinucleotide target (Lampe et al., 1998). In addition, the *Himar1* transposon system is known to yield a random distribution of insertions in genome (Nilsson et al., 2014; Withers et al., 2014). However, the usefulness of this mutagenesis system in *A. actinomycetemcomitans* has not been determined. In this study, we report the utility of the *Himar1* transposon system to perform genome wide mutagenesis in *A. actinomycetemcomitans* of different serotypes. Since biofilm formation is critical for *A. actinomycetemcomitans’* persistence in the oral cavity, in this work, we describe the genetic screen and present data that supports its use as an effective method for identifying novel genetic elements involved in the biofilm formation of *A. actinomycetemcomitans*.

## Materials and Methods

### Bacterial Strains and Culture Conditions

*Aggregatibacter actinomycetemcomitans* ATCC 700685 (strain HK1651, serotype b), ATCC 33384 (strain NCTC 9710, serotype c), ATCC 43717 (strain SUNYab 75, serotype a), ATCC 43719 (strain SUNYab 67, serotype c) were obtained from the American Type Culture Collection (Manassas). All *A. actinomycetemcomitans* strains used in this study were chloramphenicol and kanamycin sensitive. Bacteria were cultured in brain heart infusion (BHI) broth (Acumedia) and incubated at 37°C in an atmosphere supplemented with 5% CO_2_. *E. coli* 1354 (Barrett et al., 2008), a diaminopimelic acid (DAP) auxotroph, was cultured in LB broth (Acumedia) supplemented with 100 μg/mL DAP (Sigma) and incubated with aeration at 37°C.

### Transposon Mutagenesis

**FIGURE 1:**
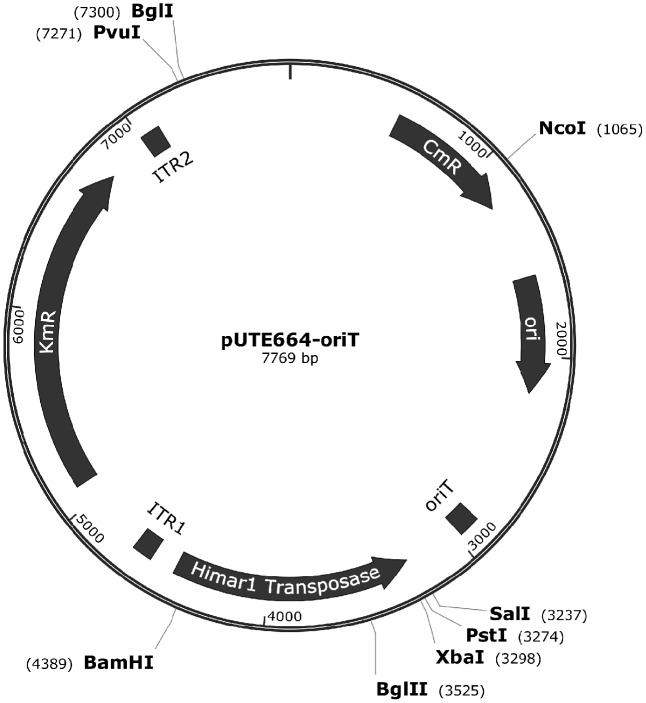
Vector map of pUTE664-oriT. This plasmid contains a functional *mariner* inverted terminal repeat (ITR) with kanamycin (Km) resistance cassette, and a backbone vector including a hyperactive *Himar1* transposase. ori, replication origin of plasmid in *E. coli*; oriT, transfer function; Km^R^, kanamycin resistant gene; Cm^R^, chloramphenicol resistant gene.

The *Himar1* transposon delivery plasmid, pUTE664-oriT (**Figure 1**), was kindly provided by Mr. Yahua Chen from the National University of Singapore. pUTE664-oriT was transformed into *E. coli* 1354 by electroporation, and selected on LB agar supplemented with 100 μg/mL DAP (Sigma) and 20 μg/mL chloramphenicol. A library of *A. actinomycetemcomitans* transconjugants was generated as follows. Overnight cultures of *A. actinomycetemcomitans* and *E. coli* 1354pUTE664-oriT were diluted 1:10 in fresh BHI and LB broth supplemented with 100 μg/mL DAP and 20 μg/mL chloramphenicol, respectively, and grown for 6 h. Conjugation was performed by mixing *A. actinomycetemcomitans* (5 × 10^8^ CFU) and *E. coli* 1354pUTE664-oriT (1 × 10^8^ CFU) and resuspending in a final volume of 50 μL fresh BHI broth with DAP (100 μg/mL), and spreading onto a piece of cellulose nitrate paper (Sartorius) placed over the surface of a BHI agar plate. Conjugation was allowed to take place for 6 h at 37°C in an aerobic atmosphere with 5% CO_2_. Bacteria were washed off from the surface of the filter paper with 1 mL BHI broth, and 100 μL of the bacterial suspension was plated on BHI selective agar supplemented with 12.5 μg/mL kanamycin, and incubated at 37°C in an atmosphere with 5% CO_2_ for 48 h. The frequency of transposition was calculated as the number of mutants on selective agars (plasmid recipient) divided by the number of *E. coli* 1354pUTE664-oriT (plasmid donor).

### Characterization of *A. actinomycetemcomitans* Transposon Mutants

To determine if the *Himar1*-based transposon has integrated into the genome of *A. actinomycetemcomitans*, each isolated single colony of transposon mutants was picked and inoculated on BHI agar supplemented with either 12.5 μg/mL kanamycin or 20 μg/mL chloramphenicol in parallel. Following successful integration of transposon into the bacterial genome, the backbone of plasmid conferring resistance to chloramphenicol resistant gene will be lost. Therefore, being resistant to kanamycin and sensitive to chloramphenicol is an indication that the plasmid has integrated into the genome. The stability of the transposon inserts was determined through serial passaging of isolated mutants on BHI agar without antibiotics for seven passages. The presence of the *Himar1* transposon cassette in the genome of these mutants was further validated by PCR. Genomic DNA was isolated from these mutants using the QIAamp DNA mini purification kit (Qiagen) according to the manufacturer’s protocol. PCR reaction consisted of 100 ng of genomic DNA, 0.5 μM each of forward and reverse primers targeting the kanamycin cassette, GoTaq Master Mix in a final volume of 20 μL. Sequences of primer used were HimarKm forward (5′-CCGGTATAAAGGGACCACCT) and reverse (5′- CAGGCTTGATCCCCAGTAAG). The PCR thermocycling consisted of an initial denaturation at 94°C for 3 min followed by 35 cycles of 94°C for 40 s, 59°C for 40 s, and 72°C for 30 s, and a final extension of 72°C for 10 min. The PCR products were analyzed on a 1.5% agarose gel.

### Southern Blot Analysis

Genomic DNA was digested with the restriction enzymes *Eco*RI (Promega) and *Bam*HI (Promega). Following digestion, DNA fragments were purified by ethanol precipitation. Purified DNA fragments were electrophoresed on a 0.8% agarose gel (BioRad). DNA was transferred to a nitrocellulose membrane (BioRad) via capillary transfer for Southern hybridization which was carried out as described previously (Sambrook et al., 1989). A 600 bp fragment of the *Himar1* transposon was amplified by PCR as described above using primers HimarKm forward and reverse. This DNA fragment was used as probe. Labeling and detection of the probe was carried out using the DIG high prime DNA labeling and detection kit (Roche) according to the manufacturer’s protocol.

### Identification of Transposon Insertion Site

To determine the site of *Himar1* insertion, genomic DNA was isolated from the mutants using the QIAamp DNA mini purification kit (Qiagen) according to the manufacturer’s protocol. DNA sequences flanking the transposon were determined by inverse PCR and DNA sequencing. Bacterial genomic DNA was digested with *Sau*3AI (Promega) and self-ligated with T4 DNA ligase (Promega), and used as template for PCR. Inverse PCR was carried out using primers H2 (5′- CCAACCTTCAAATGATTCCC) and H3 (5′- GGTACTATATAAAAATAATATGCATTTAATACTAGCG), which hybridize to the end of the kanamycin resistance gene and are oriented outward. PCR reaction consisted of 2 μL of ligation mix, 0.5 μM each of H2 and H3 primers, GoTaq Master Mix (Promega), in a final volume of 50 μL. The thermal cycling protocol used was an initial denaturation of 94°C, 3 min for 1 cycle, followed by 35 cycles of 94°C for 30 s; 57°C for 30 s and 72°C for 2 min. The PCR product was purified using a PCR purification kit (Promega) and sequenced using primer H4 (5′-TATGCATTTAATACTAGCGACG). DNA sequences obtained were analyzed by BLASTN to determine the location of transposon insertion sites.

### Screening for Mutants Defective in Biofilm Formation

A library of *A. actinomycetemcomitans* ATCC 700685 transposon mutants were generated as described above. *A. actinomycetemcomitans* biofilms were cultured in 96-well flat bottom polystyrene plates (Thermo Fisher Scientific) and incubated at 37°C supplemented with 5% CO_2_ for 24 h. Crystal violet assay was carried out to quantify the amount of biofilm formed. Planktonic cells were removed and the biofilm was washed once with sterile PBS to remove residual planktonic cells. The biofilm was fixed with methanol for 10 min, after which methanol was removed and the wells allowed to air dry. Subsequently, biofilm was stained with 1% crystal violet (Sigma) for 10 min, and excess dye was removed by washing the well with distilled H_2_O. Bound crystal violet was dissolved using 33% acetic acid (Sigma), and absorbance read at optical density 580 nm. Presumptive biofilm deficient mutants obtained from the initial screen were retested in three independent trials. Each putative biofilm deficient mutant was inoculated into BHI broth and the optical density at 600 nm was determined following 24 h incubation at 37°C in an atmosphere with 5% CO_2_. Transposon insertion sites were determined only for selected mutants which were not defective in growth but deficient in biofilm formation compared to the wild-type strain.

## Results

### *Himar1* Transposon Mutagenesis in *A. actinomycetemcomitans*

**FIGURE 2:**
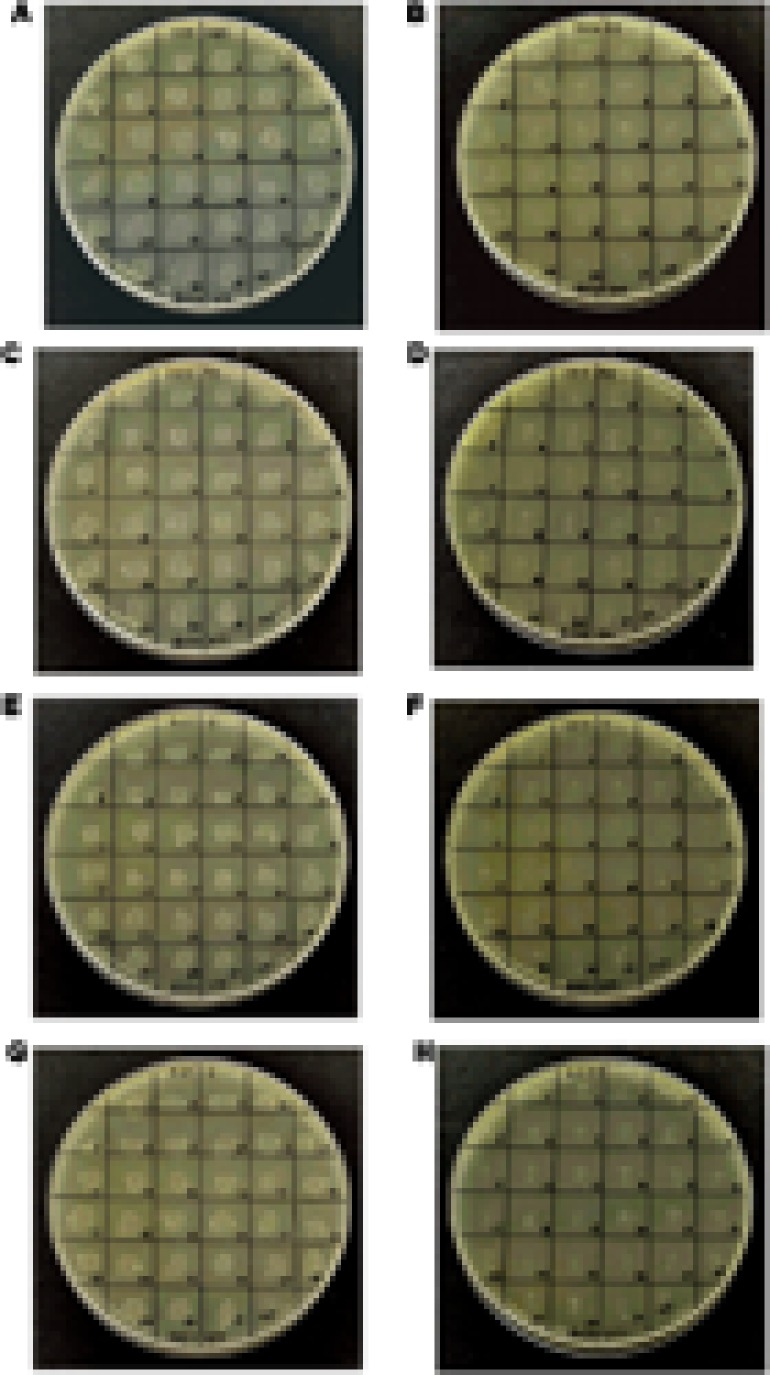
Resistance of *Aggregatibacter actinomycetemcomitans* transconjugants. Random transconjugants (*n* = 31) were selected and inoculated on either BHI agar supplemented with kanamycin **(A,C,E,G)** or chloramphenicol **(B,D,F,H)**. The resistance of *A. actinomycetemcomitans* ATCC 700685 **(A,B)**, 33384 **(C,D)**, 43717 **(E,F)**, and 43719 **(G,H)** wild-type (indicated as WT), and transconjugants to kanamycin and chloramphenicol are shown.

**FIGURE 3:**
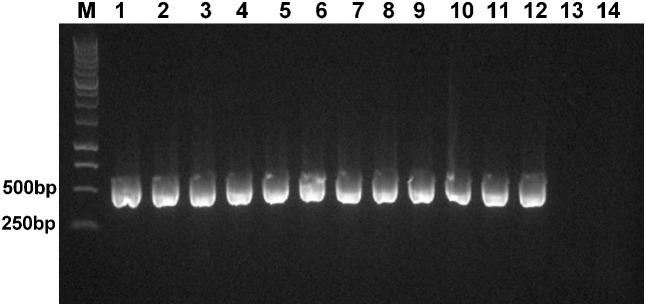
Verification of the presence of *Himar1* transposon cassette. Genomic DNA was extracted from 11 randomly picked *A. actinomycetemcomitans* transconjugants after seven serial passages. PCR was carried out using primers targeting the kanamycin resistance cassette. Lane M: 1 kb ladder (Promega); lane 1–11: randomly picked mutants with *Himar1* transposon insertions; lane 12: pUTE664-oriT (positive control); lane 13: genomic DNA of *A. actinomycetemcomitans* ATCC 700685; lane 14: no template DNA (negative control).

**FIGURE 4:**
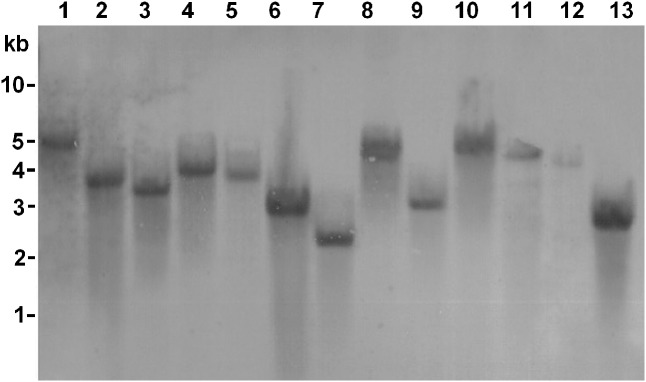
Southern blot of *Himar1* transposon insertions. Genomic DNAs were extracted from 13 random transconjugants. DNA was digested with *Eco*RI and *Bam*HI. Digoxigenin-labeled DNA containing the insertion sequence was used as a probe. The sizes of DNA markers are shown on the left in kilobases. Lanes 1–13 consisted of digested DNA from random transconjugants.

We found that *Himar1*-based transposon mutagenesis can be successfully applied in four different *A. actinomycetemcomitans* strains, belonging to serotypes a, b, and c. The transposition frequencies for all the four bacterial strains did not vary significantly at ×10^-4^, indicating that high frequency of transposition was obtained. For each strain, randomly picked transconjugants (*n* = 31) were kanamycin resistant and chloramphenicol sensitive, demonstrating that the plasmid containing the *mariner* transposon was not capable of replicating in *A. actinomycetemcomitans* (**Figure 2**). The stability of transposon inserts was determined in *A. actinomycetemcomitans* ATCC 700685 (serotype b) which is the most commonly isolated serotype from patients with aggressive periodontitis (Zambon et al., 1983). The presence of the transposon inserts was determined in 31 randomly picked transconjugants by determining their ability to grow on kanamycin resistant agar after seven passages. At the 7th passage, bacterial cells would have undergone at least 150 generations since the doubling time of *A. actinomycetemcomitans* is about 3 h (Ding et al., 2016). This is consistent with previous reports that transposon insertions are stably inherited in *A. actinomycetemcomitans* (Kolodrubetz and Kraig, 1994; Thomson et al., 1999). We found that all 31 transconjugants were able to grow on agar plates with kanamycin following serial passaging (data not shown). PCR further showed the presence of the transposon cassette in the genome of these mutants (**Figure 3**). Mutants were randomly picked and Southern blot analysis was performed to confirm single transposon insertion. All 13 mutants analyzed showed single insertion in the genome (**Figure 4**).

### Isolation of *A. actinomycetemcomitans* Mutants with Deficiency in Biofilm Formation

**FIGURE 5:**
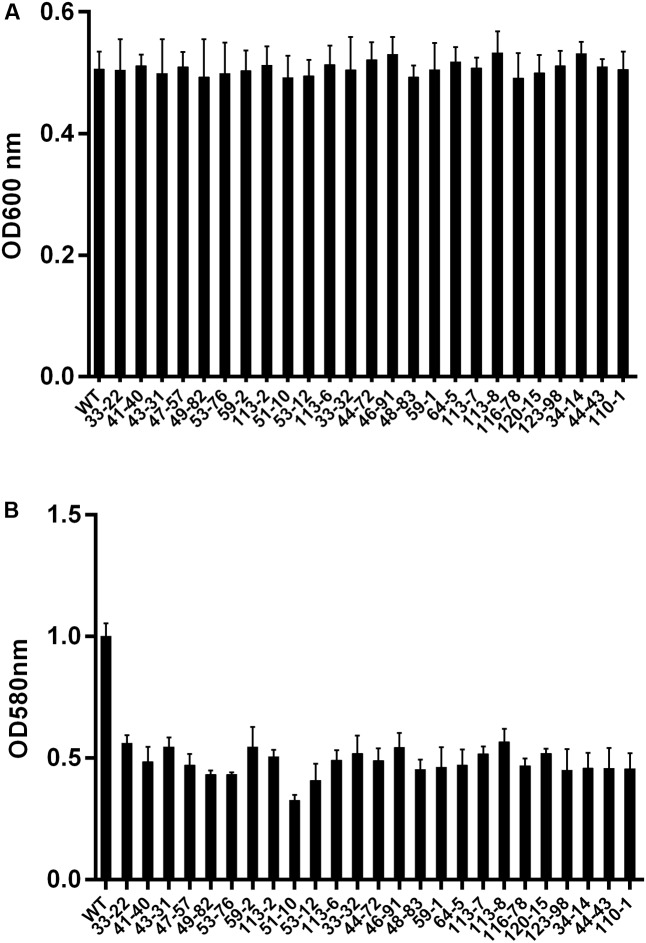
The biofilm forming ability of *A. actinomycetemcomitans* transconjugants. **(A)** Culture density of biofilm deficient mutants following overnight incubation at 37°C in an atmosphere with 5% CO_2_. **(B)** Fold differences in biomass in biofilm deficient transconjugants. Mutants were picked and cultured in 96-well polystyrene plates for 24 h, and the biomass of mutants were determined by crystal violet assay. WT: wild-type strain *A. actinomycetemcomitans* 700685.

Biofilm formation is one of the important mechanisms employed by *A. actinomycetemcomitans* to enhance their survival in the oral cavity. We employed the *Himar1* transposon insertion library to identify mutants defective in biofilm formation in *A. actinomycetemcomitans* ATCC 700685. Approximately 16,000 transposon mutants were obtained from our library of mutants, which have been verified beforehand to be kanamycin resistant and chloramphenicol sensitive. These mutants were screened for defects in biofilm formation using a microtiter plate biofilm assay. About 40% of these mutants were found to be defective in growth. As a mutation conferring a growth defect would affect biofilm formation directly or indirectly, only mutants exhibiting growth similar to that of wild-type strain were further studied (**Figures 5A,B**). A total of 25 mutants were identified to be biofilm deficient with biofilm biomass significantly lower than wild-type strain. The location of the transposon insertion sites in these mutants were determined and are shown in **Table 1**. Among the 22 mutants with transposon insertions in genes with annotated functions, eight belonged to putative membrane proteins including ion transporters, efflux pumps and secretion systems. The other 10 biofilm defective mutants possessed disruption in genes encoding putative enzymes involved in the synthesis of extracellular polymeric substance (EPS), proteins involved in bacterial metabolism and oxidative stress response, while three mutants with disruptions in transcription regulators were identified (i.e., transcriptional regulator TyrR, and transcriptional regulator LysR) to be important for biofilm formation. A mutant with transposon insertion in the pilus gene *flp-1* which has been previously reported to be essentially in adherence of *A. actinomycetemcomitans* was also identified in our screen.

**Table 1:** Transposon insertion sites in *Aggregatibacter actinomycetemcomitans* ATCC 700685 mutants with reduction in biomass.

Functional groups	Mutant No.	Gene locus^∗^	Putative function	Reduction in biomass	Previously described
Membrane proteins	33-22	CF65_00062	multidrug resistance protein	43.9%	No
	41-40	CF65_02975	integral membrane protein	51.5%	No
	43-31	CF65_02334	outer membrane protein P1 precursor	45.5%	No
	47-57	CF65_01344	magnesium transporter CorA	53.0%	No
	49-82	CF65_01341	multidrug efflux protein	56.8%	No
	59-2	CF65_02805	peptidase C39	56.8%	No
	53-76	CF65_02294	Na^+^/H^+^ anti-porter NhaC	45.5%	No
	113-2	CF65_02532	TonB-dependent receptor	49.4%	No
Gene regulation	51-10	CF65_01698	transcriptional regulator	67.5%	No
	53-12	CF65_01579	transcriptional regulator, TyrR	59.2%	No
	113-6	CF65_01794	transcriptional regulator, LysR family	51.0%	No
Metabolism	33-32	CF65_02251	biotin sulfoxide reductase	48.2%	No
	44-72	CF65_02933	sulfur acceptor protein CsdL	51.0%	No
	46-91	CF65_02631	UDP-phosphate galactose phosphotransferase	45.7%	No
	48-83	CF65_01934	histidine phosphatase	54.7%	No
	59-1	CF65_02382	glucuronate isomerase	53.9%	No
	64-5	CF65_02983	pyruvate formate-lyase	52.9%	No
	113-7	CF65_03351	tRNA-Lys	48.3%	No
	113-8	CF65_02683	electron transporter	43.3%	No
	116-78	CF65_03076	23S ribosomal RNA	53.2%	No
	120-15	CF65_00557	ATPase	48.1%	No
Other functions	123-98	CF65_00867	Flp-1	55.1%	Yes
	34-14	CF65_02545	hypothetical protein	54.2%	No
	44-43	CF65_00379	hypothetical protein	54.3%	No
	110-1	CF65_01357	hypothetical protein	54.5%	No


^∗^*Obtained from NCBI*.

## Discussion

We have demonstrated the use of *Himar1*-based transposon for random mutagenesis in *A. actinomycetemcomitans*. The transposition frequency was high at ×10^-4^ for all four strains of *A. actinomycetemcomitans* belonging to serotypes a, b, and c. This transposition efficiency was higher than the reported transposition frequency of Tn*916* and Tn*5* which is ×10^-7^ (Clewell and Gawron-Burke, 1986), and similar to the efficiency of Tn*10* and IS*903*ϕ*kan* systems. However, the Tn*10* system has preference for hotspots containing six base pair symmetrical consensus sequence of 5′-GCTNAGC-3′, while the IS*903* system requires host factors such as GTP for transposition (Coros et al., 2005). *Himar1* transposon only requires TA dinucleotides for successful insertion. This is in contrast to other transposon systems such as Tn*916* and Tn*5*, which require longer and more complex consensus motifs for insertion (Goryshin et al., 1998; Mullany et al., 2012). Analysis of the genome sequence of *A. actinomycetemcomitans* revealed that the 2.1 Mb DNA contains 1.2 × 10^5^ TA sites which could have accounted for the high frequency of transposition obtained in this study. The transposition of *Himar1* in *A. actinomycetemcomitans* genome was stable since all the mutants remained kanamycin resistant after at least 150 generations when serially passaged in the absence of antibiotic selection.

Even though substantial progress has been made in understanding the virulence of *A. actinomycetemcomitans*, it remains a challenge to study the genetics of the organism’s colonization and persistence in the oral cavity, and identify novel virulence factors due to limited genetic tools available to manipulate *A. actinomycetemcomitans*. So far, the recipient strains of *A. actinomycetemcomitans* used in transposon mutagenesis studies are limited to nalidixic acid or rifampin resistant isolates of *A. actinomycetemcomitans* (Kolodrubetz and Kraig, 1994; Thomson et al., 1999; Kachlany et al., 2000a; Isaza et al., 2008; Nunes et al., 2016). These strains are employed as these antibiotics are used for counter-selection during conjugation experiments with *E. coli*. In this work, we employed an *E. coli* DAP auxotroph as the donor so that counter-selection during conjugation is independent of antibiotic selection. Using this approach, we showed that *Himar1* transposon mutagenesis can be achieved in four different wild-type strains of *A. actinomycetemcomitans* encompassing serotypes a, b, and c which are the predominant oral isolates (Dahlén et al., 2002; Yang et al., 2005; Teixeira et al., 2006) with no resistance gene markers.

Biofilm formation is an important virulence attribute of *A. actinomycetemcomitans.* Bacteria in biofilm exhibit an increased resistance to antibiotics and killing by host defenses compared to their planktonic counterparts (Thrower et al., 1997; Fine et al., 2001). Despite the importance of biofilm formation in the pathogenesis of *A. actinomycetemcomitans*, our understanding of key genes involved in the establishment of a structured bacterial community formation is still limited. Through screening of a mutant library of 16,000 transconjugants, 25 mutants were identified to be defective in biofilm formation without significant impairment in growth compared to the wild-type. We have identified one mutant with insertion in genes previously known to be associated with *A. actinomycetemcomitans* biofilm formation, and 21 mutants with insertions in genes which have not been reported to be involved in biofilm in *A. actinomycetemcomitans* but reported elsewhere to be critical in biofilm formation in other bacterial species. The genes identified in our screen include membrane proteins, structural proteins and metabolic enzymes.

The tight adherence (*tad*) locus comprises 14 genes (*flp-1*, *flp-2*, *tadV*, *rcpCAB*, and *tadZABCDEFG*) encoding factors that are essential for biofilm formation, colonization and pathogenesis in *A. actinomycetemcomitans*. Mutations in this gene locus cause loss of adherence, co-aggregation and formation of biofilm in *A. actinomycetemcomitans* (Kachlany et al., 2000b; Planet et al., 2003; Perez et al., 2006). *flp-1* is the gene encoding the major structural component of Flp pilus (Inoue et al., 1998). In our screen, mutation of *flp-1* resulted in significant reduction in biofilm formation in *A. actinomycetemcomitans* which is in consistent with the previous findings that *flp-1* plays a key role in the pathogenesis of *A. actinomycetemcomitans.*

Our screen uncovered a mutant with disruption in putative iron transporters namely TonB-dependent receptor. We speculate that this gene is likely critical to facilitate the import of iron from the extracellular environment into the periplasmic space in *A. actinomycetemcomitans.* Levels of iron in the environment have been reported to regulate biofilm formation of *A. actinomycetemcomitans*. Under iron chelated conditions, fewer and larger aggregates form with poorly attached, weaker biofilms, compared to biofilms cultured in presence of iron (Rhodes et al., 2007; Amarasinghe et al., 2012). Several other membrane protein mutants were also discovered to be deficient in biofilm formation. These include a putative magnesium transporter and efflux pumps. Electrostatic interactions contribute to biofilm cohesion and cations such as magnesium are significant cross linkers of the biofilm matrix because they contribute to the integrity and stability of the outer membranes of the bacteria (Geesey et al., 2000). While the role of efflux pumps in biofilm formation in *A. actinomycetemcomitans* has not been established, efflux pumps have been shown to be important in the development of biofilm in *P. aeruginosa* (De Kievit et al., 2001) and *E. coli* (Kvist et al., 2008). In our screen, we identified two putative multidrug resistance proteins and a sodium-hydrogen antiporter to be important in biofilm. These proteins could support biofilm formation through clearing metabolic waste products and regulating intracellular pH, respectively. A transposon mutant of *A. actinomycetemcomitans* with insertional mutation in C39 peptidase was found to have reduced biofilm formation. This putative protein of the type I secretion family could be involved in the secretion of proteins essential in biofilm development.

Putative genes encoding several enzymes either directly involved in biofilm structure or metabolic processes were found to be involved in biofilm formation. The putative UDP-phosphate galactose phosphotransferase gene of *A. actinomycetemcomitans* could be functioning through affecting the production of EPS (Levander and Rådström, 2001; Dertli et al., 2013). EPS are biopolymers of microbial origin in which biofilm microorganisms are embedded. EPS determines the immediate conditions of life of biofilm cells living in this microenvironment by affecting adherence and mechanical stability (Flemming et al., 2007). The activity of pyruvate formate-lyase, an enzyme involved in synthesis of formate is increased in *Staphylococcus aureus* biofilm, while formate significantly increased *Campylobacter jejuni*’s biofilm formation under low oxygen tension (Leibig et al., 2011). Thus, formate might play a role in optimizing *A. actinomycetemcomitans*’s adaptation from planktonic form to biofilm. In our screen, a mutant encoding a putative ATPase was discovered to be compromised in biofilm formation. ATPases are proteins that catalyze ATP in cells and have been suggested to be responsible for stress tolerance, intracellular replication and biofilm formation in bacteria (Frees et al., 2004). The TadA protein is important in biofilm formation in *A. actinomycetemcomitans* and it has been proposed that the ATPase activity of this protein is required to energize the assembly or secretion of Flp pili (Bhattacharjee et al., 2001). However, future studies will be required to determine the functional role of the ATPase gene identified in our screen in biofilm formation.

Although our screen was relatively large, it is likely not saturating. Genes which have been reported to regulate surface associated proteins or biofilm structures of *A. actinomycetemcomitans* such as the *dspB* (Kaplan et al., 2003) and *pgaABCD* (Shanmugam et al., 2017) were not picked up in our screen. Analysis of the genome sequence revealed that there are approximately 1.2 × 10^5^ TA sites in *A. actinomycetemcomitans* genome, therefore, a sample size of at least 1.2 × 10^5^ mutants is needed to achieve a saturated mutagenesis, assuming that each mutation targets a unique site. In addition, this could have also been attributed to different bacterial strains and culture conditions employed. Interestingly, we did not pick up mutation in the same gene twice. This is an indication that *Himar*1 transposon likely occurred randomly in the genome of *A. actinomycetemcomitans* rather than having preference for specific hotspots. Although we have identified several genes associated with biofilm formation of *A. actinomycetemcomitans* through genome wide screening, these findings needs to be further verified through in-frame gene deletion and complementation studies as transposon insertions could potentially generate polar mutations.

## Conclusion

Our data demonstrated that the *Himar1* transposon is an efficient system to generate random mutants in *A. actinomycetemcomitans*. This mutagenesis system allows flexibility of carrying out mutagenesis without specific requirement for recipient strains and could be employed to identify novel essential genes for survival and virulence of this periodontal pathogen.

## Author Contributions

QD performed the experiments, analyzed the data, and drafted the manuscript. KST conceived the study, analyzed the data, and wrote the manuscript. Both authors have read and approved the final manuscript.

## Conflict of Interest Statement

The authors declare that the research was conducted in the absence of any commercial or financial relationships that could be construed as a potential conflict of interest.
